# Supplementary material for: Conditional Knockout of Hypoxia-Inducible Factor 1-Alpha in Tumor-Infiltrating Neutrophils Protects against Pancreatic Ductal Adenocarcinoma
Source: Int J Mol Sci. 2023 Jan 1;24(1):753. doi: 10.3390/ijms24010753 (PMC9821271; doi:10.3390/ijms24010753)
Supplement: Supplementary file 1 [file ijms-24-00753-s001.zip › ijms-2063386-supplementary.pdf]

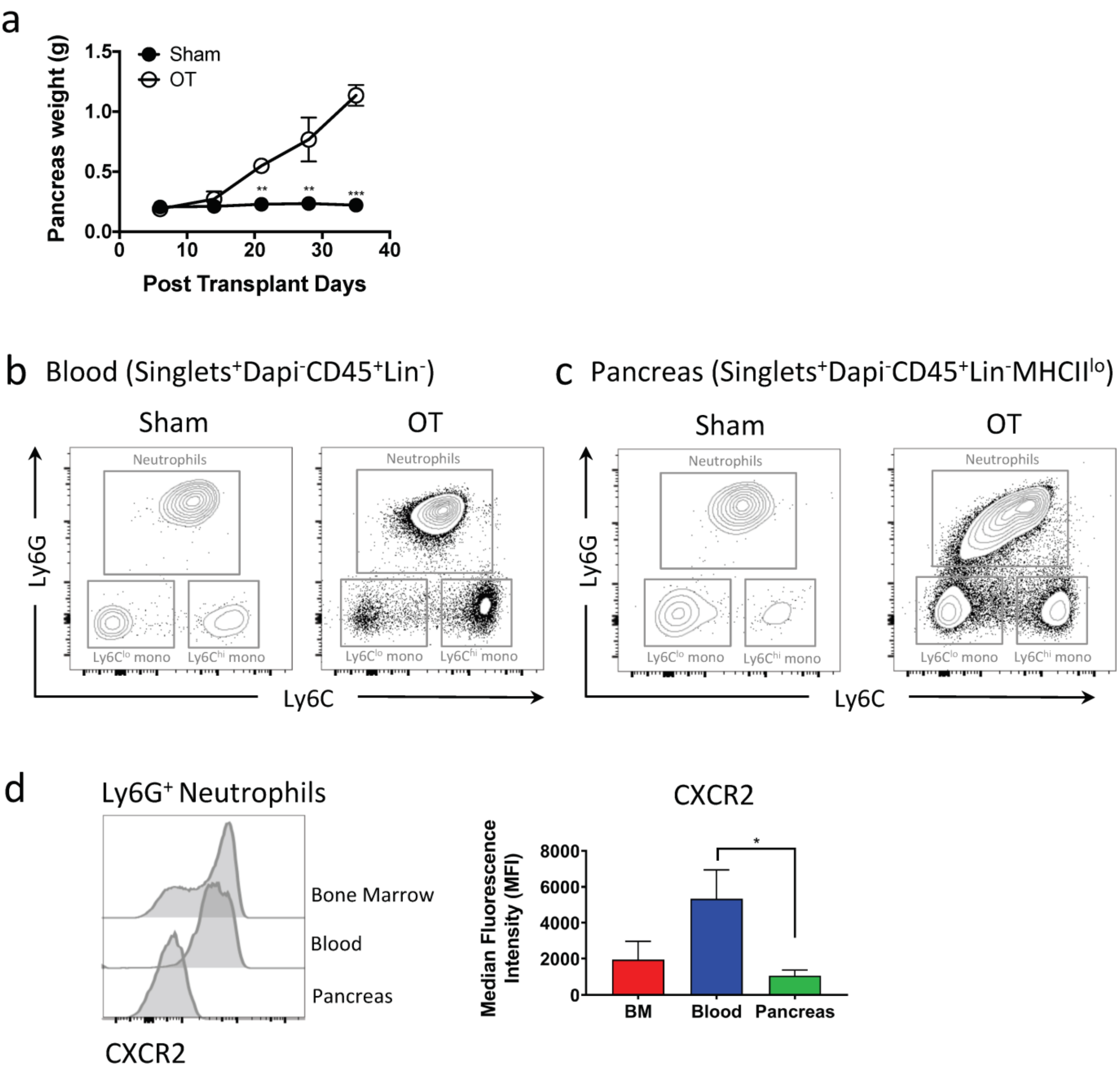

Supplementary Figure S1. (**a**) Scatter plot of orthotopic tumor weights at 6, 14, 21, 28 and 35 days post transplantation in orthotopic transplanted (OT) and sham non-tumor bearing mice (n = 3 mice per experiment at each time point). Data represent the means ± SEM, **p < 0.01; ***p < 0.001 by unpaired student's t-test with 95% confidence interval. (**b**) Flow cytometry gating strategy of blood and (**c**) pancreatic neutrophil populations in a representative OT and wild-type (WT) sample. (**d**) Representative flow cytometry histogram showing CXCR2 expression in the bone marrow, blood and pancreas of OT mice (n = 10 of three independent experiments). * p < 0.05.

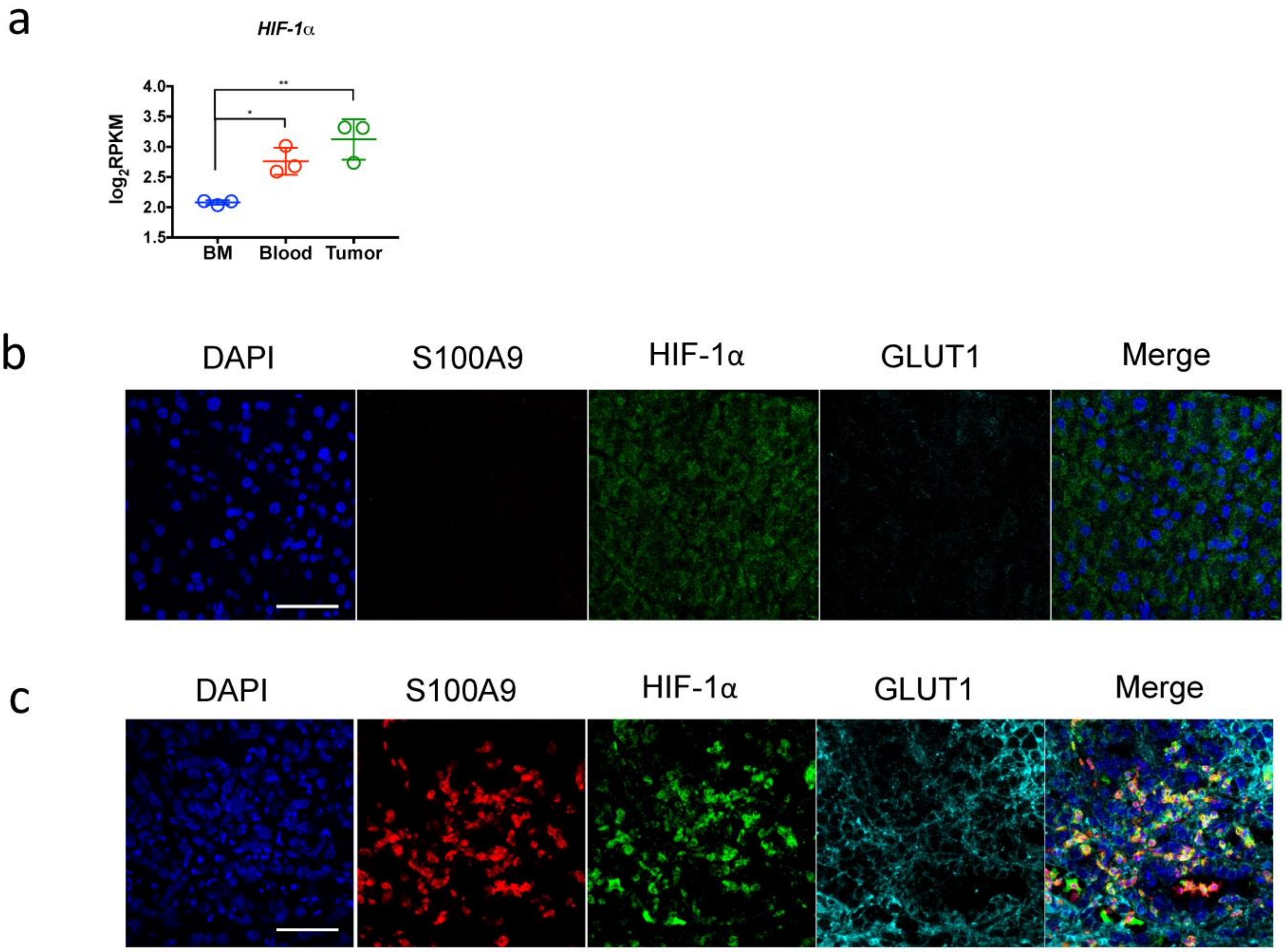

Supplementary Figure S2. (**a**) Bone marrow (BM), blood and tumor neutrophils were sorted based on their expression of Dapi^{-}CD45^{+}Lin(CD3/B220/NK1.1)$^{-}$ Ly6G^{+}Ly6C^{+} (orthotopic transplanted [OT] n = 5 mice pooled, three independent experiments). Hierarchical clustering and principal component analysis (PCA) were performed with log$_2$-transformed values. Shown are scatter plots of transcriptomic data of HIF-1α expression in the BM, blood and tumor neutrophils respectively. (**b-c**) Formalin-fixed paraffin-embedded pancreata sections of sham non-tumor bearing control mice (**b**) and orthotopic transplanted (OT) mice (**c**) stained with a neutrophil marker, S100A9 (red), and glycolytic markers HIF-1α (white) and GLUT1 (green). Immunofluorescence images are representative of n = 3 mice per group, 10 - 15 fields of view. Scale bar = 100 μM.

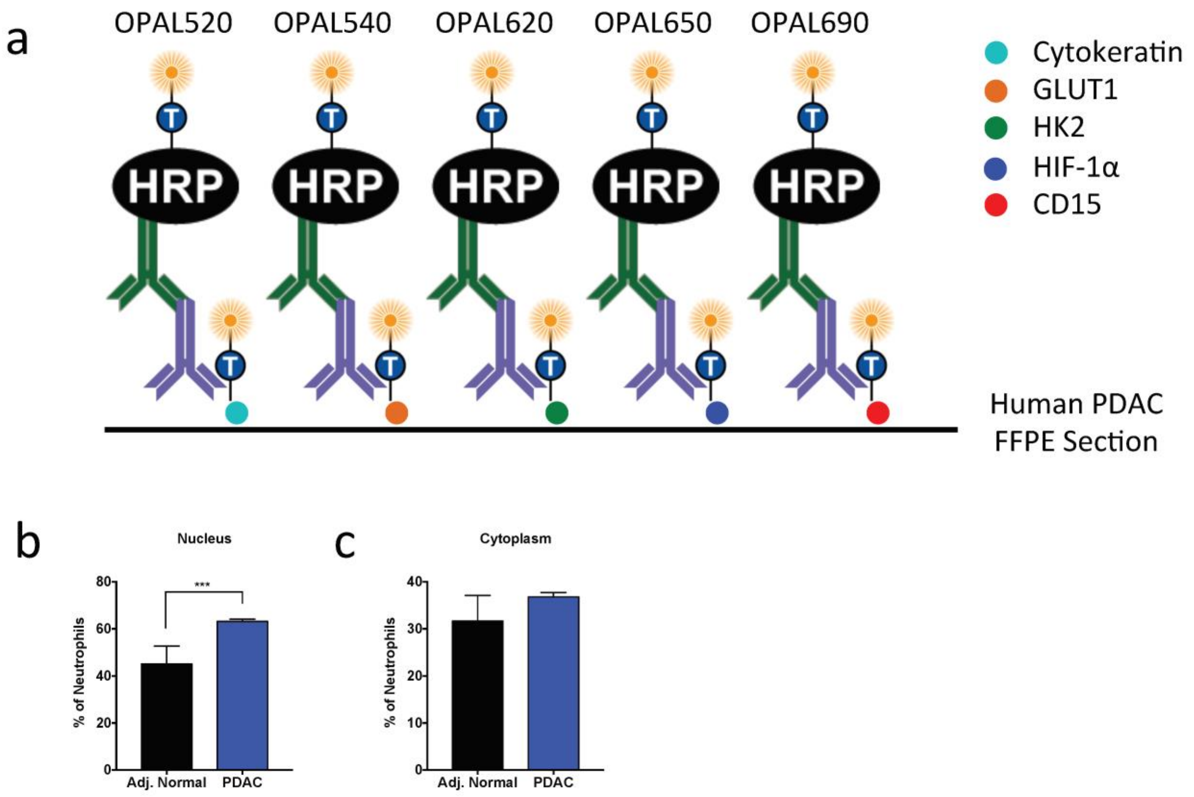

Supplementary Figure S3. (**a**) Sequential, multiplex, immunofluorescence panel of neutrophil marker (CD15), anti-pan cytokeratin (CK), and metabolic markers (HIF-1α, GLUT1 and HK2) to stain formalin-fixed paraffin-embedded (FFPE) pancreas sections obtained from multi-center PDAC patient cohorts. (**b**) Activation and translocation of HIF-1α was assessed based on HIF-1α expression in the nucleus. (**c**) Non-activated HIF-1α expression was assessed in the cytoplasm. Data represent the means ± SEM, ***$p < 0.001$ by unpaired student's t-test with a 95% confidence interval.

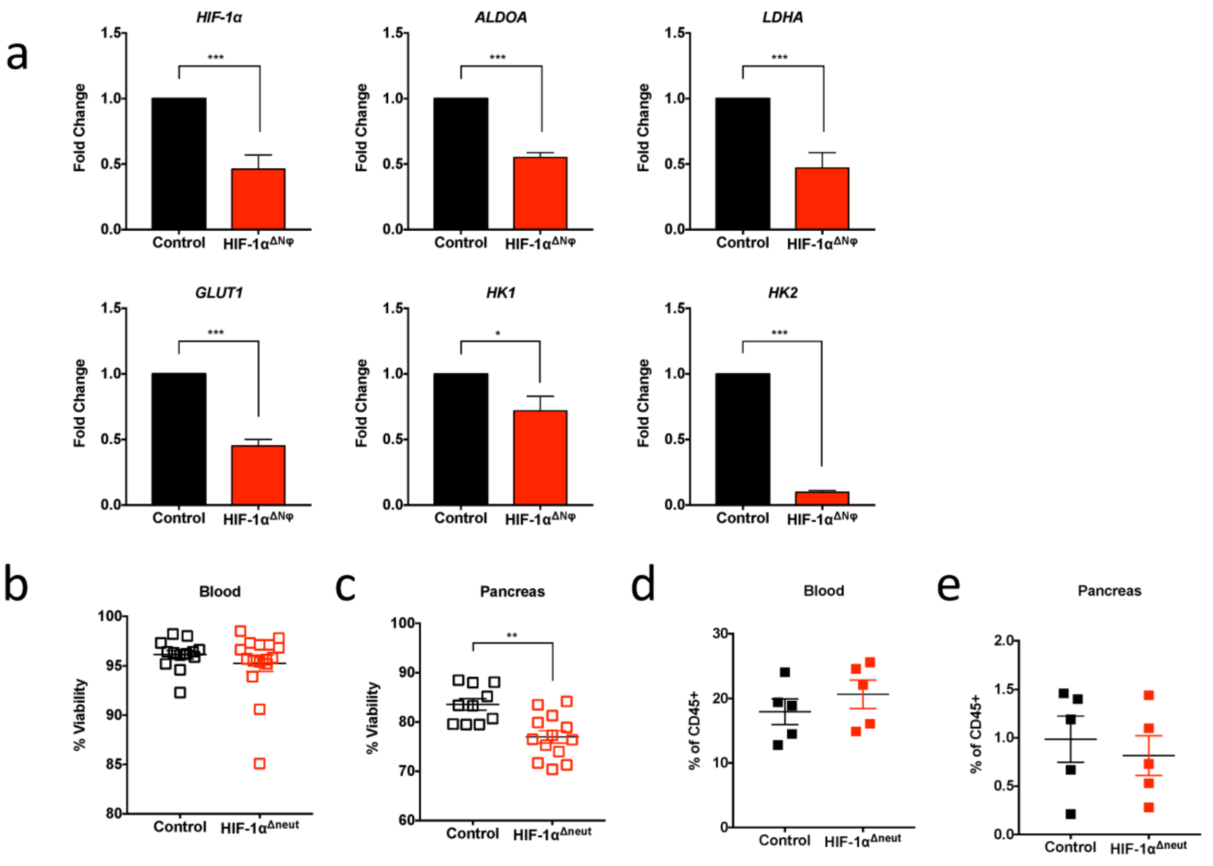

Supplementary Figure S4. (**a**) HIF-1$\alpha^{\Delta N\varphi}$ orthotopic transplanted and littermate HIF-1$\alpha^{fl/fl}$ control mice pancreatic neutrophils were assessed at the transcript level for glycolytic markers, *HIF-1α*, *ALDOA*, *LDHA*, *GLUT1*, *HK1* and *HK2* (n = 3 mice per group, three independent experiments). (**b-c**) Blood and pancreatic tumors from tumor-bearing HIF-1$\alpha^{\Delta N\varphi}$ orthotopic transplanted (OT) and littermate HIF-1$\alpha^{fl/fl}$ control OT mice were harvested at day 28 post transplantation and the myeloid cell populations were assessed by flow cytometry. The scatter plot of infiltrating neutrophils [Dapi$^-$ CD45$^+$Lin(CD3/B220/NK1.1)$^-$ Ly6G$^+$Ly6C$^+$] are plotted as a percentage of total CD45+ cells, from HIF-1$\alpha^{\Delta N\varphi}$ OT mice (red clear) and littermate HIF-1$\alpha^{fl/fl}$ controls (black clear) (OT blood n = 16, sham n = 13; OT pancreas n = 13, sham n = 10; of three independent experiments, pooled). (**d-e**) Blood and pancreas from non tumor-bearing HIF-1$\alpha^{\Delta N\varphi}$ WT mice and littermate HIF-1$\alpha^{fl/fl}$ control WT mice were harvested and the myeloid cell populations were assessed by flow cytometry. The scatter plot of infiltrating neutrophils [Dapi$^-$CD45$^+$Lin(CD3/B220/NK1.1)$^-$ Ly6G$^+$Ly6C$^+$] are plotted as a percentage of total CD45+ cells, from HIF-1$\alpha^{\Delta N\varphi}$ WT mice (red solid) and littermate HIF-1$\alpha^{fl/fl}$ WT controls (black solid) (n = 5). Data are means ± SEM, *$p < 0.05$; ***$p < 0.001$ by unpaired student's t-test with 95% confidence interval.

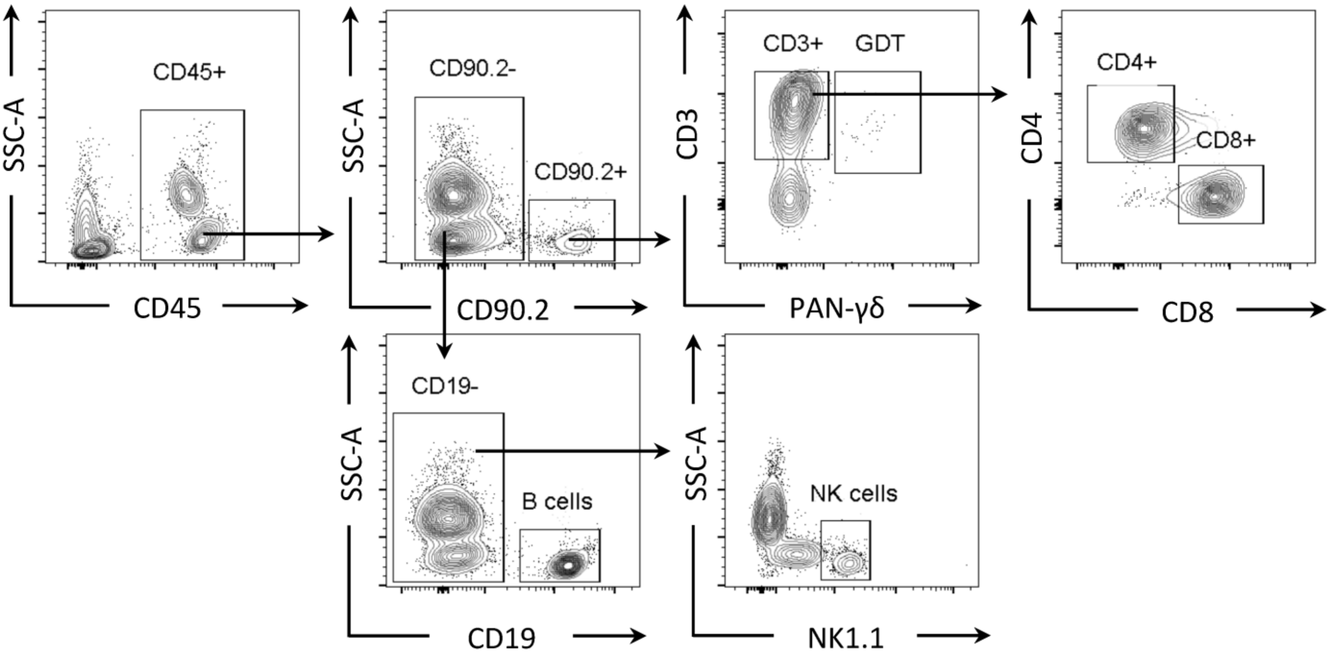

Supplementary Figure S5. Conditional knockout of HIF-1α in neutrophils promotes anti-tumoral activity via increased ROS and activation of cytotoxic T lymphocytes (CTL) and natural killer (NK) cells cytotoxicity. Neutrophils infiltrate pancreatic tumor lesions in large numbers and adopt a metabolic signature similar to that of PDAC tumor cells and the Warburg effect. These infiltrating neutrophils support tumor progression by up-regulating HIF-1α and Arg-1, thereby inhibiting anti-tumoral CTL and NK cell activity. Loss of HIF-1α by conditional knockout in neutrophils subverts the pro-tumoral activity of these neutrophils by up-regulating ROS, and increasing the production of CTL/NK cell stimulating cytokines such as IL-12 and TNF-α. This mechanism opens new avenues for potential pharmacologics that target neutrophil metabolic pathways to improve disease outcomes.

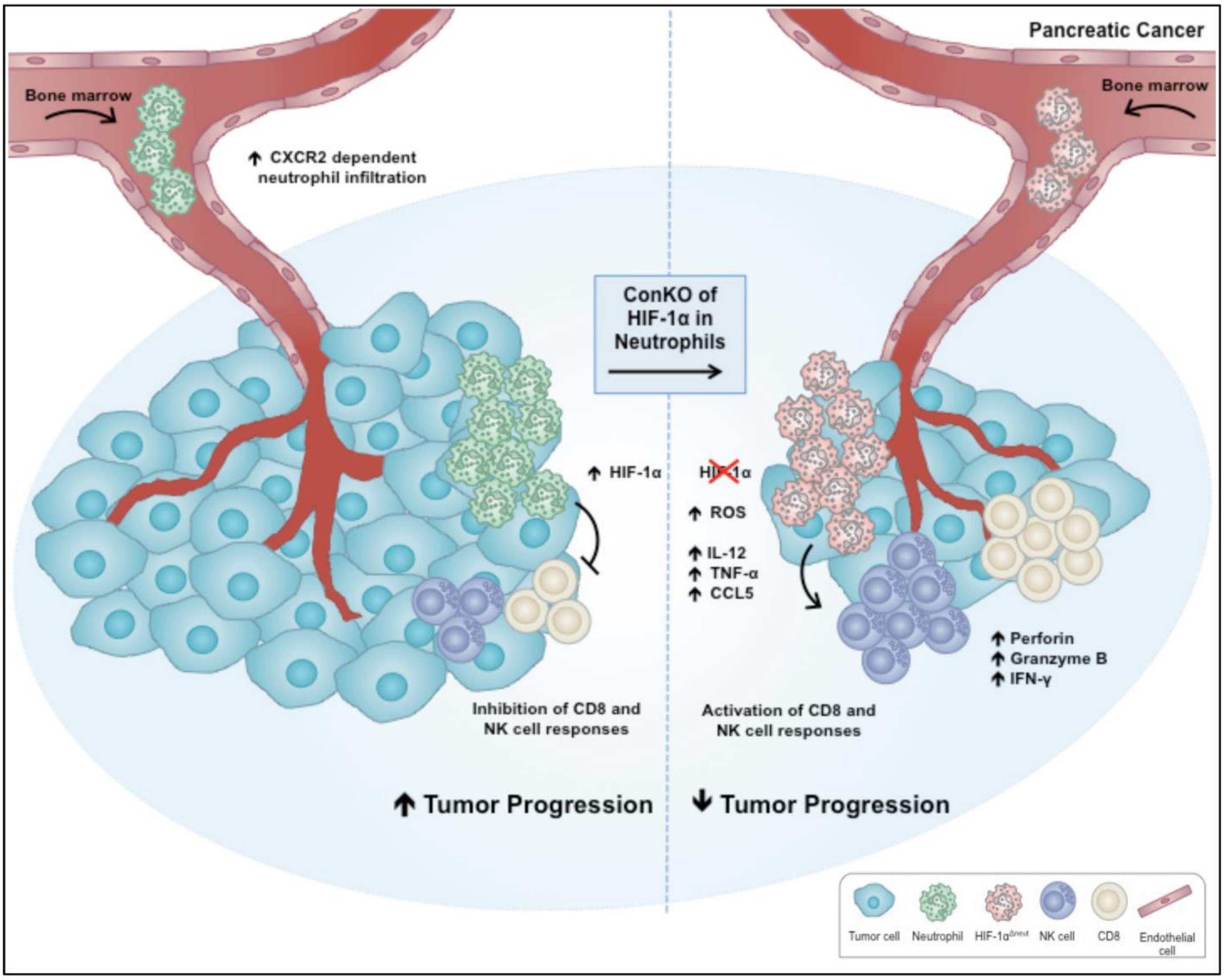

Supplementary Figure S6. Conditional knockout of HIF-1α in neutrophils promotes anti-tumoral activity via increased ROS and activation of cytotoxic T lymphocytes (CTL) and natural killer (NK) cells cytotoxicity. Neutrophils infiltrate pancreatic tumor lesions in large numbers and adopt a metabolic signature similar to that of PDAC tumor cells and the Warburg effect. These infiltrating neutrophils support tumor progression by up-regulating HIF-1α and Arg-1, thereby inhibiting anti-tumoral CTL and NK cell activity. Loss of HIF-1α by conditional knockout in neutrophils subverts the pro-tumoral activity of these neutrophils by up-regulating ROS, and increasing the production of CTL/NK cell stimulating cytokines such as IL-12 and TNF-α. This mechanism opens new avenues for potential pharmacologics that target neutrophil metabolic pathways to improve disease outcomes.

Supplementary Table S1. Mouse primer sequences used for RT-qPCR

Name of Primer	Sequence (5' to 3')
**CCL2 – Forward**	CAGGTCCCTGTCATGCTTCT
**CCL2 – Reverse**	GTCAGCACAGACCTCTCTCT
**CCL3 – Forward**	ACCATGACACTCTGCAACCA
**CCL3 – Reverse**	TCAGGCATTCAGTTCCAGGT
**Arginase1 – Forward**	GATTGGCAAGGTGATGGAAG
**Arginase1 – Reverse**	TCAGTCCCTGGCTTATGGTT
**IL-12 – Forward**	CGTGCTCATGGCTGGTGCAAAG
**IL-12 – Reverse**	GAACACATGCCCACTTGCTG
**CCL5 – Forward**	ACCATGAAGATCTCTGCAGC
**CCL5 – Reverse**	TGAACCCACTTCTTCTCTGG
**HIF-1α – Forward**	GTCGGACAGCCTCACCAAACAG
**HIF-1α – Reverse**	TAGGTAGTGAGCCACCAGTGTCC
**ALDOA – Forward**	AGAACACCGAGGAGAACAGG
**ALDOA – Reverse**	AGTTGTCTCGCCATTGGTTC
**LDHA – Forward**	GTGTAACTGCGAACTCCAAGC
**LDHA – Reverse**	TGGATTGGAGACGATCAGCAG
**GLUT1 – Forward**	CATCCTTATTGCCCAGGTGTTT
**GLUT1 – Reverse**	GAAGACGACACTGAGCAGCAGA
**HK1 – Forward**	TGCCATGCGGCTCTCTGATG
**HK1 – Reverse**	CTTGACGGAGGCCGTTGGGTT
**HK2 – Forward**	AGCTGTTTGACCACATTGCC
**HK2 – Reverse**	CACGCCACTGGACTTGAAC
**β-actin – Forward**	AGAGGGAAATCGTGCGTGAC
**β-actin – Reverse**	CAATAGTGATGACCTGGCCGT
